# Clinical Remission of Cutaneous Squamous Cell Carcinoma of the Auricle with Cetuximab and Nivolumab

**DOI:** 10.3390/jcm7010010

**Published:** 2018-01-10

**Authors:** Alessandra Chen, Nabilah Ali, Peter Boasberg, Allen S. Ho

**Affiliations:** 1 Samuel Oschin Comprehensive Cancer Institute, Cedars-Sinai Medical Center, Los Angeles, CA 90048, USA; aychen@mednet.ucla.edu (A.C.); Nabilah.Ali@csmns.org (N.A.); pboasberg@theangelesclinic.org (P.B.); 2 David Geffen School of Medicine at University of California, Los Angeles, CA 90095, USA; 3 Department of Surgery, Cedars-Sinai Medical Center, Los Angeles, CA 90048, USA; 4 The Angeles Clinic and Research Institute, Cedars-Sinai Medical Center, Los Angeles, CA 90048, USA

**Keywords:** cutaneous squamous cell carcinoma, cetuximab, nivolumab

## Abstract

Cutaneous squamous cell carcinomas (SCC) affecting the regions of the head and neck can be challenging to resect surgically and refractory to chemotherapy or radiation therapy. Consequently; the treatment of squamous cell carcinomas of the skin is a focus of current research. One such advancement is immunotherapy. Herein we describe clinical remission of invasive, poorly differentiated squamous cell carcinoma of the pre-auricular region with external auditory canal involvement using cetuximab, an epidermal growth factor receptor (EGFR) antibody; and nivolumab, a programmed death receptor-1 (PD-1) antibody. Such durable and comprehensive disease resolution demonstrates the therapeutic potential of cetuximab and nivolumab in surgically challenging, treatment-resistant cutaneous squamous cell carcinoma.

## 1. Introduction

Squamous cell carcinoma (SCC) of the skin affects an estimated 700,000 individuals each year [1]. Metastatic disease is rare, occurring in approximately 1.9–2.6% of cases [2]. However, mortality for squamous cell carcinoma that is locally advanced or involves lymph nodes is significantly greater [3,4,5]. Other factors including diameter exceeding 20 mm, poor differentiation, location on the lip, ear, or temple, invasion beyond subcutaneous fat, or perineural invasion contribute to an increased risk of death [6]. Given the functional considerations and aesthetic sensitivity of these regions, aggressive surgical treatment, which is first-line therapy for cutaneous SCC of the head and neck, often prove particularly difficult. We present a case of treatment-refractory, invasive squamous cell carcinoma located in the pre-auricular space and external auditory canal (EAC) that was successfully salvaged with cetuximab (Erbitux, Lilly, Indianapolis, IN, USA), an epidermal growth factor receptor antibody, and nivolumab (Opdivo, Bristol-Meyers Squibb, New York, NY, USA), a programmed death receptor-1 (PD-1) antibody. Resolution of disease with this novel immunomodulatory regimen spared the patient an extensive and potentially disfiguring surgical operation.

## 2. Case Report

### 2.1. Patient History

A 74-year-old Caucasian male, a former lifeguard with a history of substantial sun exposure, presented with a left progressively enlarging pre-auricular cutaneous lesion. He had a past medical history significant for arteriosclerotic heart disease, renal failure on peritoneal dialysis, and multiple cutaneous cancers which include melanoma of his right calf and left and right lower back, in addition to SCC and basal cell carcinoma of his scalp, face, and extremities. He had first noticed the lesion approximately one year earlier. The mass was surgically excised and found positive for invasive SCC. In addition, the patient underwent radiation therapy. The lesion appeared to regress two months after completion of radiation. However, three months later, his cancer recurred and exhibited rapidly progressive growth.

**Figure 1:**
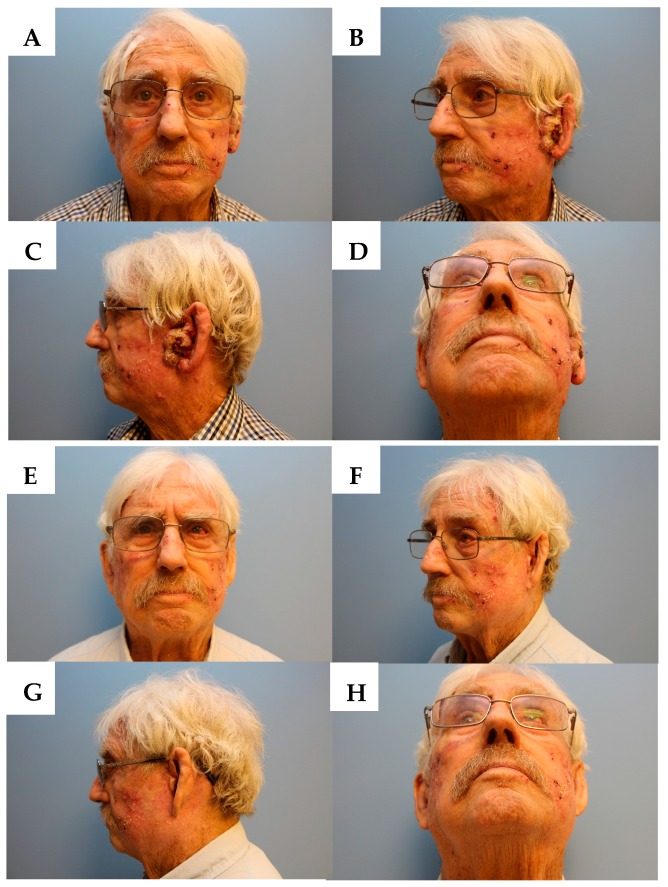
(**A**) Frontal view before immunotherapy. (**B**) Left oblique view before immunotherapy. (**C**) Left lateral view before immunotherapy. (**D**) Base view before immunotherapy. (**E**) Frontal view after immunotherapy. (**F**) Left oblique view after immunotherapy. (**G**) Left lateral view after immunotherapy. (**H**) Base view after immunotherapy.

**Figure 2:**
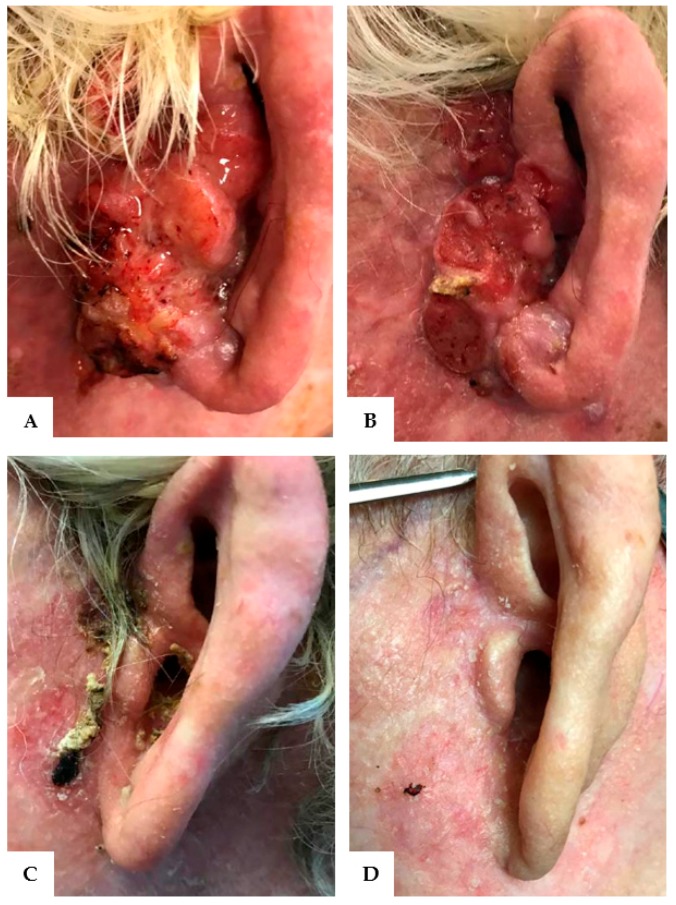
Resolution of invasive squamous cell carcinoma of the left pre-auricular region and external auditory canal after treatment with cetuximab and nivolumab. (**A**) Tumor at maximal growth. (**B**) One month after initiation of immunotherapy. (**C**) Three months after initiation of immunotherapy. (**D**) Complete remission at six months.

Physical exam revealed severely sun-damaged skin of the head and neck. The left pre-auricular area had a 5 cm × 3 cm exophytic cutaneous mass which obliterated the pretragal region and encroached into the concha bowl, the left EAC, and the left lobule (Figure 1A–D and Figure 2A). Once the EAC was debrided, the tympanic membrane and the proximal EAC was found to be intact and without tumor involvement. The remainder of the exam, including facial nerve function, was unremarkable.

### 2.2. Decision-Making and Treatment Course

**Figure 3:**
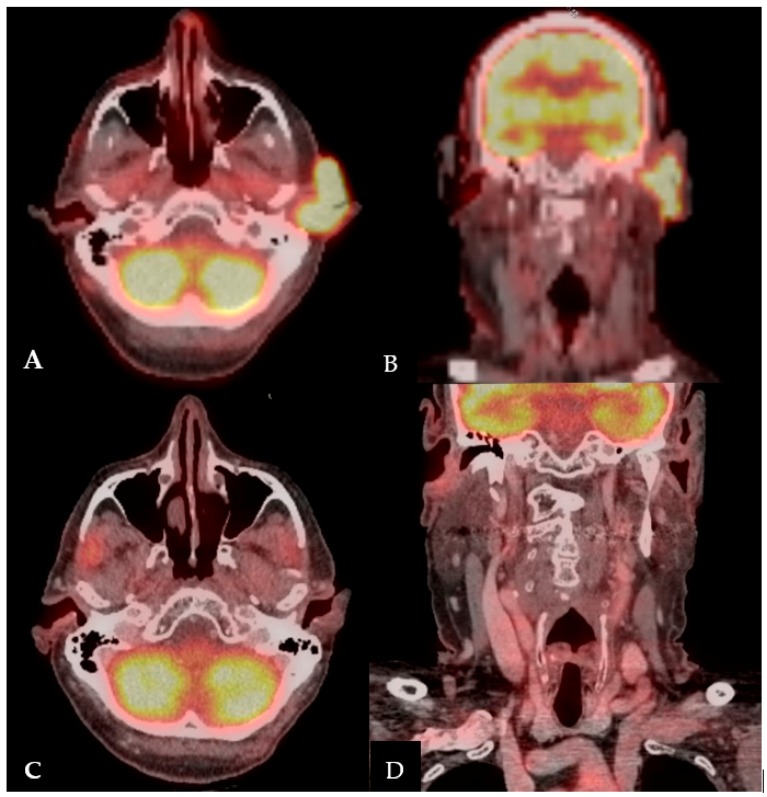
Positron Emission Tomography/Computed Tomography: (**A**) Axial view before immunotherapy. (**B**) Coronal view before immunotherapy. (**C**) Axial view eight months after initiating immunotherapy. (**D**) Coronal view eight months after initiating immunotherapy.

Given the treatment-resistant nature and progressive enlargement of his left pre-auricular SCC, the patient was referred to Head and Neck Surgery for discussion of resection. Magnetic resonance imaging (MRI) of his brain demonstrated focal hypo-intensity in the right parietal calvarium, corresponding to an area of increased fludeoxyglucose (FDG) uptake on Positron Emission Tomography/Computed Tomography (PET/CT) (Figure 3A–B). This finding raised suspicion for possible calvarium involvement. Multidisciplinary tumor board discussion led to the recommendation for definitive surgical intervention, including total auriculectomy, lateral temporal bone resection, superficial vs total parotidectomy, left modified radical neck dissection, possible craniotomy, and free flap reconstruction. This surgical plan would involve Head and Neck Surgery, Neurosurgery, and Plastic and Reconstructive Surgery teams, with high risk of facial nerve paralysis. The patient would require an auricular prosthesis postoperatively.

Given the extensiveness of the operation, the patient declined radical surgery and elected for a multi-agent nivolumab and cextuximab regimen. He was started on cetuximab weekly and nivolumab biweekly. He required a treatment break from cetuximab secondary to the development of cutaneous toxicity, which has been suggested to be a proxy for response to treatment. Otherwise, he tolerated consistent infusions of biweekly nivolumab (Figure 2B).

### 2.3. Clinical Outcome

After one month of immunotherapy, the SCC had stopped progressing and after three months, the mass had demonstrated remarkable regression (Figure 2B,C). Within six months, the patient was in complete remission, with no evidence of squamous cell carcinoma on his left pre-auricular region or external auditory canal (Figure 1E–H and Figure 2D). After eight months of immunotherapy with nivolumab, PET/CT revealed the resolution of the previously seen intense activity mass in the left pre-auricular region extending to the inner ear (Figure 3C,D). After one year of continued treatment with nivolumab, the patient remains free of disease recurrence.

## 3. Discussion

In this case, complete remission of invasive, poorly differentiated squamous cell carcinoma of the ear was achieved through an immunotherapy-based regimen. Despite the patient’s other co-morbidities, the combination of cetuximab, an antibody against epidermal growth factor receptor (EGFR), and nivolumab, an antibody against programmed death receptor-1 (PD-1), was well-tolerated by the patient. Remarkably, clinical regression of the pre-auricular mass was achieved within six months. The patient subsequently avoided extensive surgery, potential facial nerve paralysis, and the need for an auricular prosthesis.

The standard of care for resectable squamous cell carcinoma normally entails surgical extirpation followed by radiation [7]. Because the patient declined surgery and failed prior radiation therapy, immunotherapy was offered. Immunotherapy is not a well-established treatment for cutaneous SCC. This case notably demonstrates a promising role for combination cetuximab and nivolumab therapy in squamous cell carcinomas that may have metastatic involvement, are refractory to chemotherapy, or are in regions difficult to surgically resect. To our knowledge, this combination has not been well studied in cutaneous SCC. Induction therapy with both cetuximab and nivolumab may have been key to the remission of this patient’s tumor. With continued nivolumab maintenance therapy, the patient remained in remission without adverse effects.

Regarding the efficacy of cetuximab, the expression of EGFR is known to be increased in cutaneous SCC and is purported to contribute to tumor proliferation [8]. Response to cetuximab has been shown to correlate with higher EGFR expression. In addition to the inhibition of EGFR, cetuximab activates antibody-dependent, cell-mediated cytotoxicity and stimulates the development of cytotoxic lymphocytes, resulting in the immune-mediated destruction of tumor cells [9]. A phase II clinical trial of cetuximab as first-line therapy in patients with unresectable SCC of the skin found 58% of patients achieved stable disease [10]. Only 8% had a partial response and 3% (one patient) had complete remission. With respect to nivolumab, this agent is an immune checkpoint inhibitor, which prevents the deactivation of T lymphocytes and preserves immune cell function. Nivolumab is an Food and Drug Administration-approved treatment modality for recurrent or metastatic SCC of the head and neck and for advanced melanoma [11,12,13]. However, a study on the role of nivolumab in non-melanoma SCC of the skin has not yet been comprehensively studied.

The synergistic effect of cetuximab and nivolumab likely involves immune cell stimulation in combination with the prevention of immune suppression. Cetuximab-mediated activation of cytotoxic cells induces a negative feedback loop of immunosuppressive activity, thus paradoxically limiting the action of cetuximab. It has been hypothesized that when administered with cetuximab, immune checkpoint inhibitors, such as nivolumab, are able to prevent this immunosuppressive response [9]. Blockade of the interaction between programmed death receptor 1 and its ligand (PDL-1) disrupts the action of regulatory T and myeloid-derived suppressor cells, enabling cetuximab-induced immune cells to survive [9]. The mechanism underlying combination treatment with cetuximab and nivolumab may also involve the EGFR-mediated upregulation of PDL-1 [14]. Disruption of the stimulatory pathway for PD-1 via the blockade of EGFR, in concert with direct antagonism of the PD-1 receptor, may inhibit the deactivation of cytotoxic T lymphocytes. Thus, the addition of nivolumab to cetuximab therapy may preserve immune cell action against tumor cells. Numerous clinical trials examining cetuximab in combination with other immune checkpoint inhibitors are ongoing [9].

Recently, reports of six patients have demonstrated tumor regression of treatment-refractory cutaneous SCC specifically affecting the pre- and post-auricular and temporal regions with nivolumab as well as pembrolizumab, another PD-1 antibody [15,16,17,18,19]. Similar to nivolumab, pembrolizumab exerts its effect by interrupting the pathway that disarms T lymphocytes, preventing the immune-mediated destruction of tumor cells. Review of the literature revealed 12 cases of locally advanced or metastatic SCC treated with nivolumab or pembrolizumab [15,16,17,18,19,20,21,22]. An ongoing clinical trial (NCT02964559) is examining pembrolizumab as a primary treatment for locally advanced or metastatic SCC of the skin [23]. One case also demonstrated near-complete regression without recurrence with REGN2810, a PD-1 inhibitor. In an ongoing clinical trial (NCT02383212) investigating REGN2810 in unresectable, locally advanced, or metastatic cutaneous SCC, 10 of 26 patients have maintained a response for eight to more than 40 weeks [24].

Although the efficacy of immunotherapy can be impressive and durable, overall response rates quoted in the literature remain low, at approximately 10–25% [25]. The selection of patients most likely to benefit from immunotherapy remains a pivotal concern, and the indications for immunotherapy, such as PDL-1 positivity or PD-1 expression, continue to evolve. Research has indicated that the expression of PD-1 may be increased in infiltrative cutaneous SCC and in cutaneous SCC with perineural invasion [22]. For certain sites such as head and neck, lung, and melanoma, PD-L1 positivity >1% has been reported to predict response, yet this has not been consistent [26]. Moreover, a lack of PD-L1 positivity does not preclude response. Similar results have been found with high mutational load. More recently, immunotherapy use has been approved for recurrent/metastatic solid tumors independent of site; required criteria include high microsatellite instability (MSI-high) or mismatch repair-deficiency (MMR-deficient) [12]. Notably, the molecular sequencing profile of the tumor in this case was negative for PD-L1 and MSI-stable, though the mutational load was high (123 mutations/megabase). Such varied results paired with the impressive clinical response demonstrate the pressing need to define accurate predictors of response.

Other biomarker mutations identified in this case included ATM, BRCA2, CDK4, cMET, FH, KMT2C, NF2, NOTCH1, PDGFRA, TP53. Whole exome sequencing of aggressive cutaneous SCCs identified 11 potential drivers of tumor growth [27]. Our patient expressed three of those mutations; KMT2C, NOTCH1, and TP53. Notably, KMT2C was associated with worse outcome and greater bone invasion. Investigation of the correlations between patient mutations expressed in metastatic or recurrent SCC of the skin and patient responses to PD-1 inhibition may help to identify which patients may benefit from immunotherapy. In addition, targeted sequencing of lymph node metastases in cutaneous SCC has also revealed several pathways involved in pathogenesis, including activating mutation of the epidermal growth factor receptor pathway, which is inhibited by cetuximab [28].

This report is limited in its scope, as we describe only one patient. The outcome of this case does not predict the efficacy of nivolumab in other cases of aggressive cutaneous SCC. We are unable to apply these findings to other patients, as the severity of disease, tolerance of adverse effects, and biological profile of the tumor will vary. There is not yet robust evidence to definitively support the use of immunotherapy over surgical resection, which is the current standard of care. Indeed, months after resolution of the mass and while still on immunotherapy, the patient developed other small scalp cutaneous malignancies that were safely excised.

Philosophically, these results are of significance to clinical decision-making in the management of resectable cutaneous malignancies. Surgical resection followed by adjuvant radiation, the current standard of care, provides higher cure rates and a more predictable treatment course, but is potentially extensive and morbid. Immune-targeting regimens have not been thoroughly investigated, and it would be irresponsible to offer such regimens as a primary modality given current data. Nonetheless, for informed patients facing substantial morbidity and who refuse surgery for justifiable reasons, this case offers a glimpse of organ-preservation therapy that is potentially curative without adverse sequelae. Further investigation is required to select patients and tailor treatment to the aggressiveness of the disease.

## 4. Conclusions

Dual treatment with cetuximab and nivolumab resulted in complete remission of an invasive, rapidly progressive cutaneous squamous cell carcinoma of the pre-auricular space and external auditory canal. Further studies are necessary to properly select patients, for an immunotherapeutic approach that may significantly impact clinical decision-making and avoid surgical morbidity in high-risk patients.
